# Self-reported use of technology by orientation and mobility clients in Australia and Malaysia before the COVID-19 pandemic

**DOI:** 10.1177/02646196211019070

**Published:** 2023-01

**Authors:** Lil Deverell, Jahar Bhowmik, Abdullah Al Mahmud, Bee Theng Lau, Fakir M Amirul Islam, Suku Sukunesan, Chris McCarthy, Denny Meyer

**Affiliations:** Swinburne University of Technology, Australia; Swinburne University of Technology, Malaysia; Swinburne University of Technology, Australia

**Keywords:** Assistive technology, Australia, blind, low vision, Malaysia, orientation and mobility

## Abstract

Since the 1960s, many electronic travel aids have been developed for people with low vision or blindness to improve their independent travel skills, but uptake of these specialist devices has been limited. This study investigated what technologies orientation and mobility (O&M) clients in Australia and Malaysia have, use, like, and want to support their travel, to inform technology research and development. This two-phase mixed-methods study surveyed O&M clients face-to-face in Malaysia (*n* = 9), and online in Australia (*n* = 50). Participants managed safe walking using a human guide, long cane, or guide dog when their vision was insufficient to see hazards, but a smartphone is now a standard travel aid in both Australia and Malaysia. Participants relied on smartphone accessibility features and identified 108 apps they used for travel: for planning (e.g., public transport timetables), sourcing information in transit (e.g., GPS location and directions, finding a taxi), sensory conversion (e.g., camera-to-voice, voice-to-text, video-to-live description), social connections (e.g., phone, email, Facebook), food (e.g., finding eateries, ordering online), and entertainment (e.g., music, games). They wanted to ‘carry less junk’, and sought better accessibility features, consistency across platforms, and fast, reliable, real-time information that supports confident, non-visual travel, especially into unfamiliar places.

## Introduction

Worldwide, there are at least 2.2 billion people who have low vision or blindness (World Health Organization, 2019). Many specialist assistive technologies have been developed to support their independent travel, but apart from the long cane, uptake of these devices has been limited (Cuturi et al., 2016). Wider consultation is needed to ensure that new technologies for travel meet the real rather than assumed needs of end-users.

Mobility is a fundamental human right (Logan et al., 2018) and embraces any activity within an environment, moving between environments, and manipulating objects (World Health Organization, 2002). A social model of disability proposes that disability is not inherent in a person but results from interactions between an individual and his or her environment (World Health Organization, 2002) and technology can help to mediate these interactions. This is evident in the way that previously able individuals and workplaces were initially disabled by ‘stay at home’ and ‘social distancing’ requirements during COVID-19 lock-downs. Some individuals and businesses will not survive COVID-19, but many have learned to work, shop, and play in a virtual world, to build relationships, manage business, and stay connected in the cloud (Jones, 2020).

Referral criteria for orientation and mobility (O&M) services differ between providers and might include vision and/or mobility problems. Broadly, O&M can embrace any whole-body action we undertake in the course of the day, regardless of visual status (L. Deverell, 2019), and most can take their O&M for granted. However, O&M services were established in Australia in 1971, and afterwards in Malaysia (Neustadt-Noy & La Grow, 2010), to equip people with low vision or blindness to move in any environment as independently as possible. This meant teaching skills for blind mobility, including long cane and guide dog travel. Today, an estimated 90% of Australian O&M clients have low vision (Ah Tong et al., 2015), and so low vision aids and visual efficiency training are commonly included in O&M services (Barraga & Collins, 1979; Shah et al., 2018). Many O&M clients also have health problems, communication challenges, intellectual disability, acquired brain injury, physical limitations, or mental illness that limit their travel.

During travel, low vision or blindness can double the time and distance taken to achieve a travel task (E. A. L. Deverell, 2016), limiting confidence, mobility, roaming range, and life-space. Low vision can cause uncertainty about objects and social encounters. Non-visual travel is tiring, often frustrating, and it is easy to get disorientated. As O&M technologies are developed to reduce these limitations, end-user engagement is needed to ensure these efforts are fit for purpose (Boninger et al., 2012; Giudice & Legge, 2008).

### O&M assistive technologies

O&M aids range from basic to sophisticated, differing in complexity, purpose, function, specificity, and price.

In O&M parlance, a long cane and a guide dog are primary mobility aids, used to detect steps and hazards ahead to prevent falls and serious injury. The long cane extends the traveller’s reach and offers some body protection. It has been in continual demand for nearly 80 years, is used worldwide, and retails at less than AUD$40 (Vision Australia, 2019). Sometimes a long cane is used in conjunction with a human guide or other physical mobility aids such as prosthetics, functional electrical stimulation, wearable exoskeletons, walking aids, wheelchairs, and motorised mobility scooters (Cowan et al., 2012).

Electronic travel aids are secondary aids, not intended to replace a long cane or guide dog, but they provide additional information during travel. Development of specialist electronic aids began in the 1960s with the Laser Cane for obstacle and landmark detection. These aids have been variously categorised as mobility aids or orientation aids; obstacle detectors or environmental imagers; offline and online personal devices; and environmental modifications (Roentgen et al., 2008). A current example is the Victor Reader Trek, a multi-purpose orientation aid for blind travellers incorporating GPS, audio-directions, and an audio book player, retailing at AUD$1000 (Humanware, 2018).

Electronic orientation aids inform navigation so that wayfinding is more fluent and precise. A compass or tactile map require good spatial cognition, whereas talking signage, GPS with audio-directions, or Bluetooth beacons do not require mental mapping skills (Giudice & Legge, 2008). This distinction is important, because although most travellers have good spatial cognition, 15% of people have difficulty with mental mapping, regardless of their visual status (E. A. L. Deverell, 2016). Spatial relationships such as angles, distances, and directions do not make much sense to them, and so they use non-spatial navigation strategies such as sequenced word instructions.

Other specialist assistive technologies for O&M include sensory translation aids that convert camera to speech, light, or soundscape, and many embellishments to the long cane. There are implantable visual prostheses, a tongue-placed electro-tactile device (TED), CASBlip converting visuals to audio, a Radio Frequency Identification Walking Stick (RFIWS) detecting the curb-line, the Cognitive Guidance System (CG System), obstacle avoidance using auto-adaptive thresholding or haptics and a laser rangefinder, Silicon Eyes, the Mini-Radar, SmartCane, RecognizeCane, K-Sonar Cane, iSONIC, Tom Pouce, Télétact, CyARM, EyeCane, and Kinect Cane (Ayton et al., 2014; Cuturi et al., 2016; Elmannai & Elleithy, 2017; Finger et al., 2016; Sahoo et al., 2019). There are aids that offer indoor and/or outdoor coverage, daytime and/or night time feedback, varying ranges of preview (<1 m to >5 m), and an ability to detect static and/or dynamic objects in the environment (Elmannai & Elleithy, 2017).

### Technology uptake and end-user engagement

The technology lifecycle moves quickly, many prototypes are never commercialised, uptake of most specialist devices remains low (Cuturi et al., 2016), and not much is known about the acceptance of O&M technologies by blind users. It is unclear whether low uptake is due to poor design and lack of consultation with end-users, lack of investment, high unit cost in a small viable market, or the potentially limiting role of O&M specialists as gatekeepers to specialist technology. Complex devices can be overwhelming (Cuturi et al., 2016) and there can be a chasm between innovators/early adopters and majority/laggards in embracing new technologies (Rogers, 2003). This chasm exists among Australian O&M specialists, with one-third in 2018 self-identifying as technology laggards, and 48% acknowledging that they need more technology skills (L. Deverell et al., 2020). A 2008 photograph shows a blind guide dog traveller – an early adopter, hungry for environmental information – slung with five different electronic travel aids each serving a unique function (Giudice & Legge, 2008). The challenge was to combine these separate functions into a single device, and now we have smartphones.

The commercial viability of specialist O&M technologies seems threatened by the rise of mainstream technologies with accessibility features. An evidence base is needed to ensure that research and development of O&M technologies is agile and appropriately targeted to end-users. Historically, O&M clients were engaged to test already-developed specialist prototypes (Smith & Penrod, 2010), but the shift towards co-design (Layton, 2012; Lee, 2014; Sanders & Stappers, 2008) has been evident in developing tactile maps (Gardiner & Perkins, 2005), investigating wheelchairs, walkers, prostheses and power mobility (Auger et al., 2015; Brandt et al., 2010; Fomiatti et al., 2014; Walsh & Petrie, 2016; Wang et al., 2013), transport access (Wong, 2018), and the safety concerns of guide dog handlers (L. Deverell & Meyer, 2016).

During 2018, the multi-disciplinary O&M research team at Swinburne University of Technology investigated the self-reported technology experiences of O&M clients and O&M professionals, to inform the development of useful O&M technologies. These parallel studies conducted before COVID-19 provide a marker in a rapidly changing technology environment. The COVID-19 pandemic has radically increased use of communication technologies, but reduced spatial mobility and public transport use (de Haas et al., 2020). Our outcomes relating to O&M professionals are published elsewhere (L. Deverell et al., 2020). This article focuses on O&M client outcomes: (1) identifying technologies that O&M clients in Australia and Malaysia have, use, like, and want to support their travel, and (2) generating ideas for O&M technologies that serve end-users’ needs.

## Materials and methods

This grounded theory, constructivist study involved a mixed-methods, two-phase survey design with a qualitative priority. A pilot technology survey for O&M clients was administered face to face in Malaysia alongside observation of technology use during travel, then the survey was revised before being administered online in Australia. Informed consent was given verbally during face-to-face interviews, and implied by online participants. The project was approved by the Swinburne University Research Ethics Committee (2016/316) and conducted in accordance with the Declaration of Helsinki.

### Survey development

Survey questions were drafted by our multidisciplinary research team in collaboration with Australian O&M clients then piloted (*n* = 2). The questions were translated into Bahasa Malaysia and back to English using Google Translate, then checked by a Malay-speaking member of the research team (S.S.). The survey form included both languages to support communication during interviews (see Supplemental S1). After reviewing the Malaysian data, the questions were expanded, with more free-text responses. The Qualtrics survey was uploaded, re-piloted (*n* = 2) and then administered online.

### Recruitment and data collection

In early 2018, an Australian O&M specialist (L.D.) spent a month in Malaysia (Kuala Lumpur and Kuching) to investigate the cultural relevance of two new functional vision and O&M assessment tools called VROOM and OMO (L. Deverell et al., 2017). During this field trip, the intentionally homogeneous convenience sample (*n* = 9) was handpicked by staff from three Malaysian blindness services. Interviews and functional assessments were conducted either at these blindness organisations or at participants’ homes. All interviewees spoke some English, but friends, family members, service providers, and colleagues assisted with interpretation when necessary.

In mid-2018, the Qualtrics survey link was distributed via email to Australian O&M service providers and support/interest groups relating to low vision or blindness, but respondents (*n* = 50) were not required to live in Australia. The survey was open for 10 weeks.

### Data analysis

Malaysian survey data were entered manually into Excel, and the Australian online survey data were exported to Excel from Qualtrics. We included all participants in the Australian cohort regardless of location assuming they all encountered the survey through the Australian contacts used for recruitment. Missing data were checked and descriptive statistics were generated using IBM SPSS Version 25 (J.B.). Complex free-text responses were unravelled in Excel with compound items separated out in column A and named in column B. The A-Z function was used to repeatedly regroup data and develop relevant categories (L.D.). We analysed descriptive statistics in conjunction with qualitative data, and then discussed interpretations with members of Swinburne’s O&M client reference group.

## Results

Proportions of people with no light perception, very low vision (with a pension or allowance) and more vision were similar for the two cohorts (Table 1). The Malaysian interview cohort (*n* = 9; 89% male; mean age: 33, age range: 20 to >70) all used English for technology, but each spoke at least one other language (Chinese, Bahasa Malaysia). The online survey cohort (*n* = 50, 42% male; mean age: 39, age range: <10 to >70) primarily lived in Australia (92%), so we refer to this as the Australian cohort although one participant lived in Canada, one in Malaysia and two in unspecified places.

**Table 1.** Visual status, literacy preferences, and devices used by Malaysian and Australian orientation and mobility clients.

		Malaysian cohort (*n* = 9) % (*n*)	Australian cohort (*n* = 50) % (*n*)
Visual status	No light perception	22 (2)	20 (10)
	Low vision, eligible for pension/allowance	67 (6)	66 (33)
	Low vision, not eligible for pension/allowance	11 (1)	8 (4)
	Full vision		6 (3)
Literacy formats	Regular print on paper	67 (6)	24 (12)
	Large print on paper		26 (13)
	Handheld low vision aids		32 (16)
	Text on screen		42 (21)
	Screen magnifier/zoom	78 (7)	36 (18)
	Screen reader/voiceover	33 (3)	46 (23)
	Voice recorder device/app	44 (4)	24 (12)
	Audio/radio		24 (12)
	Braille	33 (3)	22 (11)
	Help from other people	44 (4)	28 (14)
	Other: Google home smart speaker, Seeing AI		12 (6)
Mainstream devices	Landline telephone		42 (21)
	Mobile phone	100 (9)	84 (42)
	Tablet	22 (2)	54 (27)
	Laptop computer	78 (7)	78 (39)
	Desktop computer	22 (2)	54 (27)
	Personal activity monitor	0	24 (12)
Specialist devices	Portable braille note taker	44 (4)	14 (7)
	Standalone optical character recognition	22 (2)	4 (2)
	CCTV	33 (3)	18 (9)
	Standalone GPS	0	4 (2)
	Handheld sonar	0	10 (5)
	Sonar built into another device	0	2 (1)
	Barcode reader	0	4 (2)
	Other: electronic magnifier, Victor Stream	11 (1)	8 (4)

Two-thirds (67%) of the Australian cohort completed the survey independently but one-third had assistance, citing insufficient vision, deafness, accessibility problems, and poor technology skills. Nine parents responded on behalf of their children (two aged below 10 years, and seven aged 10–19 years); and 88% *only* used English for technology; the remainder also used Chinese, Indonesian, or German.

### What do people have? Technologies for travel and information

We asked about mobility aids and transport systems used in the past year (Table 2) to understand travel contexts for using personal devices (Table 1). Participants could choose more than one option in these multiple-choice questions.

**Table 2.** Use of mobility aids and transports by Malaysian and Australian orientation and mobility clients.

		Malaysian cohort (*n* = 9), %	Online cohort (*n* = 50), %
Mobility aids	Long cane	44	54
	Sighted/human guide	56	40
	Identification/symbol cane		22
	Guide dog	0	18
	No aids		16
	Support cane/walking stick		6
	Other: manual wheelchair, bundu basher, glasses, monocular, iPhone camera, pacer poles, wheelie walker, Lazarino, Moovit	11	2 (each)
Transports	Private car	67	96
	Bus	56	82
	Train		80
	Taxi	78	60
	Uber		38
	Aeroplane	56	60
	Tram		44
	Uber		38
	Ferry/water taxi		26
	Bicycle/tricycle/tandem	11	20
	Cruise ship/boat		14
	Motor bike		8
	Foot scooter/skateboard/skates	11	6

In Malaysia, there was greater reliance on human guided travel, including affordable taxis and rideshares. Australian participants used a wider range of personal mobility aids including guide dogs, and they travelled more commonly in a private car or used public transport systems (buses, trains, airlines, and water transports) that require independent travel skills and involve e-timetables.

A larger proportion of the Malaysian cohort read text on paper, screen magnifier, or braille, while use of regular print, audio output, and a tablet or iPad was more evident in Australia.

All Australian participants aged between 20 and 70 years used a mobile phone (64% specifying iPhone and 17% specifying Samsung); abstainers included six children and two elderly participants. All Malaysian participants used a mobile phone (44% specifying iPhone) and said most people they knew had a smartphone, but few could afford specialist O&M technologies (e.g., Trekker, Miniguide).

### What do people use? Apps, websites, and technology skills

Between them, Malaysian (67%) and Australian (86%) participants identified 108 specific apps or websites used to support their travel. These were sorted into eight categories: administration, sensory translation, personalised public transport, journey planning, travel booking, social networks, food, and entertainment (see Supplemental S2).

Malaysian participants learned many technology skills from other O&M clients, and all could access basic technology training, devices, and support through their local low vision/blindness agency.

Australian participants rated their own skills: 44% said they were good at technology and 26% helped others to use it; 32% had enough technology skills to keep them going but 26% needed more; only 8% said they used technology but did not enjoy it. Participants learned through trial and error (70%), family and friends (68%), online help (40%), colleagues (32%), a paid expert (22%), formal training or short courses (14%), and other options (22%). Several people relied on their partner to access technology and 14% needed help with installation, setup, and developing independent technology skills.

### What do people like? Technologies preferred and abandoned

All Malaysian participants looked for ease of use, clear layout, and speed in apps. In the Australian cohort, ease of use was also a priority (42%), specifically screen clarity, visual contrast, helpful instructions, consistent formatting, and useful content. Size, weight, and portability were important to 16% who wanted to ‘carry less junk’.

In the online survey, free-text comments about accessibility (32%) valued audio instructions/voiceover. Individual participants also liked braille display linked with phone for deaf-blind users; pinch and zoom functions; shaking the phone to repeat the last instruction; blue tooth connectivity; headphones enabling the traveller to hear traffic sounds; distance and facing direction to the next destination; and design for colour blindness.

Accuracy and reliability (including up-to-date data and battery life) were important to 26% of people exploring unfamiliar places; 10% valued live, timely information to keep them moving efficiently; only 4% emphasised using technology to plan, source information, and prevent travel stress. Other priorities were affordability (12%), availability (2%), and age appropriateness (2%).

Over one-quarter (28%) of online participants identified devices or technologies for travel they had but did not use, including the Buzzclip, Trekker Breeze, and apps: HereWeGo, Ariadne GPS, Sendero Guide Dogs NSW/ACT, Google maps, AppleMaps, NextThere, Moovit, TransPerth, and PTV. Reasons included insufficient phone memory, ‘clunkiness’, lack of real-time data, and confusing presentation. One person was anxious about BeMyEyes because of ‘who I might get to help me’.

### What do people want? Suggestions for developing O&M technologies

There was a call (26%) for improvement in the accessibility of current devices and apps, with a simpler, easier interface and universal inclusion of voice instructions, audible responses, and alerts that do not rely on vision. Individual participants also wanted better Android apps; more reliable GPS coverage, updated maps, and accurate synching so that information in unfamiliar places can be trusted; maximising contrast in screen design; better development of optical character recognition to check distant or high signage; greater accuracy in identifying colours and reading prices in shops; standard Bluetooth connectivity; and headphones that also allow the traveller to hear traffic sounds. ‘It is good if shops on the left are announced in the left channel of headphones, shops on the right appear in the right channel, things straight ahead are centred through both headphones (e.g., Microsoft Soundscape)’.

In managing traffic decisions, several wanted UK Neatebox products (*Welcome* and *Button* apps) in Australia and there was a proposal that electric cars, which can be difficult to hear, send information to a phone app indicating direction of travel, speed, and distance.

On public transport, ‘I want to see buses moving along Google maps showing me the way from my location to my destination’. Others wanted accessible apps announcing approaching buses and trains, and mandatory announcements on all public transport about the next stop, saying what side of the train to disembark. Access to rideshare services (e.g., Uber) was limited with ultra-low/no vision because it was difficult to identify and track the car on the smartphone screen.

Participants wanted easier navigation of both outdoor and indoor spaces, with Bluetooth beacons around public transport, universities, and shopping centres; enhanced GPS accuracy taking you ‘right to the door’ and to shop counters; ‘information in places we can find it, like stair handrails’; and Smartwatch maps combining audio and tactile/haptic information:

I want to point my phone at a shop to identify the place. If the GPS cannot identify place, I would like ability to label that place myself by pinning the place and typing a name using an accessible map within the app. Once labelled, my label on map can be shared with other users.

One person suggested that smart glasses have a lot of potential, while another wanted Siri to use left and right rather than north, south, east, west directions. An international traveller had found tactile tiles in the Netherlands that ‘make a different sound (perhaps also texture) for alerting us to where we can find information or help’.

**Figure 1.**
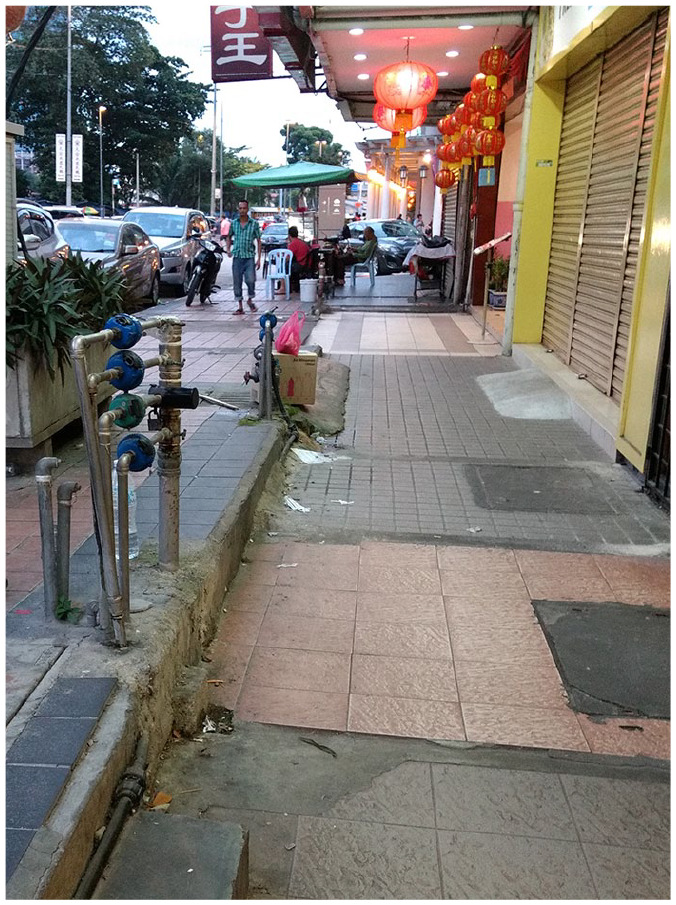
Fragmented footpath in Kuala Lumpur. Raised utility pipes, steps, ramps, planter boxes, street furniture, litter, frequent surface changes, and a motor bike parked on the footpath in Kuala Lumpur present travel challenges for a pedestrian with low vision or blindness. Photograph by L.D. Used with permission.

Individual Malaysian participants said, ‘first we need to improve facilities’; prioritise security features; and source good, affordable white canes. Malaysia needs to be ‘better mapped for walking GPS’ identifying changes in ground-plane, hazards, steps, drains and curb drops, which can be a foot deep in places (Figure 1). Malaysian participants also suggested door access software and a queue movement detector.

## Discussion

This study confirmed that uptake of specialist O&M technologies by O&M clients is low, and a smartphone is now a standard travel aid in both Australia and Malaysia. O&M clients in this study included people of all ages, a few with full vision and some who read regular print – a reminder that O&M services are not limited to blind people; low vision can take many forms, and people with full vision can have mobility problems that benefit from O&M services (Blasch et al., 2010) and O&M technologies.

The priority for participants was timely access to information *during* travel. O&M clients in Australia and Malaysia integrated diverse literacies, travel systems, mobility aids, electronic devices and apps to facilitate safe, effective, efficient travel. They used a primary mobility aid, or not, to negotiate hazards, but they used their information technologies to organise their lives, plan ahead, navigate in transit, connect with others, find food, and assuage tedium on a journey.

### Some differences between Australian and Malaysian contexts

**Figure 2.**
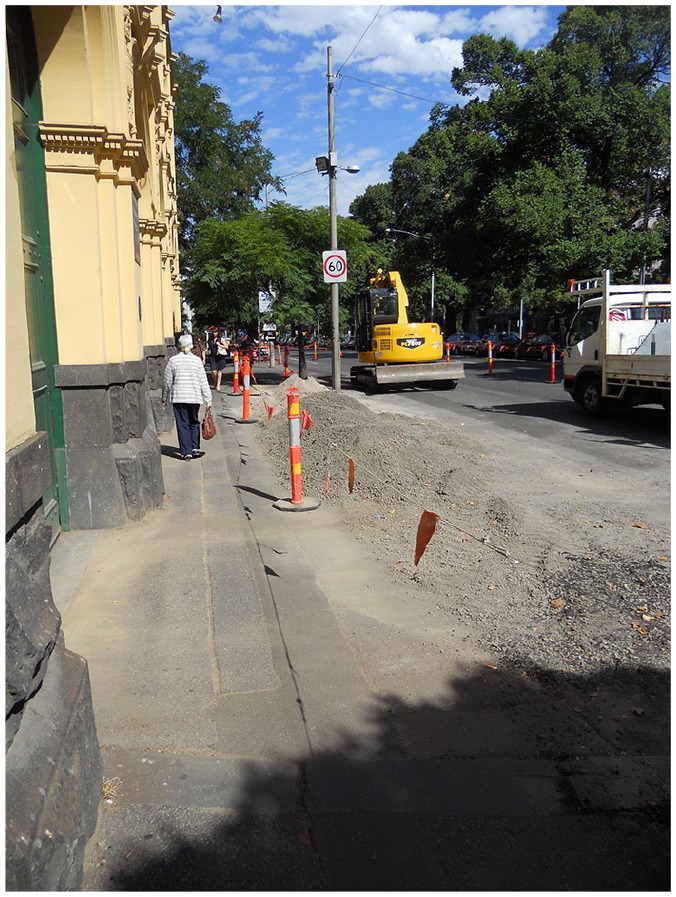
Clear pedestrian footpath barricaded from roadworks in Melbourne. Photograph by Dean Johnson. Used with permission.

Attitudes to independence, integrity of the travel environment, and population density were significant points of difference between Australian and Malaysian findings. Australians value their autonomy, and Australian standards and legislation for urban design aim for a ‘continuous accessible path of travel’ for pedestrians (Figure 2) (Sheppard, 2015). GPS technologies supported independent travel for Australian participants, especially in unfamiliar places. This is good because, with only 3 people/km^2^ in Australia compared to Malaysia’s 99 people/km^2^ (Worldometers, 2019), solo Australian travellers can have difficulty finding someone to help. However, Australian participants reported that internet coverage could be unreliable in the cities, and even more so in rural and remote areas.

With less investment in physical infrastructure, Malaysian footpaths are not designed for fluent pedestrian travel and tend to be poorly maintained. Some Malaysian participants preferred to walk on the road; more people used guided travel, or taxis and rideshare services; and hazardous footpaths made wheeled mobility difficult (e.g., walkers, wheelchairs). Conversely, Malaysia’s high population density meant that accompanied or social travel is more feasible; participants could find assistance, and internet connectivity seemed a lesser problem.

### Functional implications of available technologies

The wide-ranging uptake of mainstream smart technologies is significant for several reasons. First, diverse apps address five universal elements of travel – getting your bearings, checking groundplane, wayfinding, recognising moving parts, and finding things – measured with the VROOM and OMO functional assessment tools (L. Deverell et al., 2017). Journey planning apps were useful for getting your bearings, wayfinding, and finding things, while sensory translation apps were important for checking ground-plane, recognising moving parts, and finding things (see Supplemental S1).

Second, recognition of steps or down-curbs has presented an on-going challenge for technology developers. While electronic sensors can typically detect obstacles and up-curbs with high reliability, drop-offs and voids require more sophisticated analysis, so their detection using sensor technologies can be variable (Giudice & Legge, 2008). Live description apps like Be My Eyes and Aira now provide an alternative to wearable sensors, giving the traveller temporary access to a human guide to scan for hazards and describe the environment as needed.

Third, the low uptake of specialist aids for obstacle detection confirms adults’ opinions from our previous studies (E. A. L. Deverell, 2016; L. Deverell & Meyer, 2016) that O&M safety is more complex than simply avoiding collisions. Smart technologies are needed to navigate unfamiliar places and avoid getting lost; to deal with unexpected circumstances, silent vehicles, and inclement weather; and to interpret and navigate anxiety-provoking social challenges in transit.

Fourth, there is a high rate of depression associated with vision loss (Horowitz, 2004) which can reduce life-space and affect travel confidence, energy for unfamiliar places, and social opportunities. Smart technologies evidently address these problems: they facilitate spontaneous and planned connections with other people, increasing social reach; and they reduce the frustration and fatigue of unknowing – the travel guesswork that is cumulatively exhausting. However, O&M technologies need to deliver accurate, accessible, reliable information about the immediate environment to instil travel confidence.

Apps for social networking, food and entertainment did not separately target orientation or mobility, but they did facilitate vision- and mobility-related wellbeing (L. Deverell et al., 2017) with reading, activities, and social engagement that made tedious travel more interesting.

There were confident early adopters in the Australian cohort suggesting that there are plenty of O&M clients able to train and support Australian novices – O&M clients and professionals – in using travel technologies. This training needs to include lifestyle and entertainment apps; strategies to manage information overload, concentration, and divided attention; how to distil, store, and retrieve useful information; and couples training, so that sighted supporters can also learn accessibility features and the O&M client has backup at home.

### Implications for designing new technologies

Our results showed that participants in both Australia and Malaysia were not looking for embellishments to the long cane, or more specialist assistive devices. Rather, they appreciated and looked for continuous improvement of mainstream technologies, with universal design and specific features that provide access for all (Rose et al., 2005).

Malaysian participants demonstrated remarkable fluency in moving between visual, audio and tactile modes, and different mobility and transport systems. They valued this multi-sensory, multi-modal access to information (New London Group, 1996; Round Table, 2019) emphasising that consistency and streamlining in design help to make travel technologies and the travel environment easier to use. Along with elders and tourists, they crave ‘a seamless approach covering all links of the mobility chain . . . with focus on pedestrian navigation and public transport’ (Koutny & Miesenberger, 2015, p. 440).

Smart technologies for travel are dependent on streamlined environmental design and efficient communication networks. In Malaysia, smooth paths seem a priority for investment, whereas participants did not seem obstructed by loss of internet connection. In Australia, installation of beacons into public transport systems and public places seems to be the next step in O&M technology investment (Dunn, 2017; Merrick, 2016), while fragile or patchy connectivity was still frustrating.

Our study highlighted that GPS for drivers can get a pedestrian most of the way there, but an O&M client needs fast, reliable, real-time information and more contextual details about the pedestrian environment to avoid groundplane hazards like overflowing drains, to support safe road crossings, access public transport in a timely way, navigate indoor and outdoor spaces fluently, and locate doorways and destinations with greater precision, dignity, efficiency, and confidence. This means improving optical character recognition for interpreting text in the wild and other precise landmark features; technology for depth perception; expanding use of beacon technologies; and easier ways to record and access personalised route information.

Early adopters spoke enthusiastically about Aira and the future of live, remote assistance technologies. One participant’s reluctance to use Be My Eyes might be due to personality, social inhibitions, or perhaps a suspicion of tricking (E. A. L. Deverell, 2016). The issue of user-trust in assistive technologies has been identified (Avila et al., 2016) and warrants further consideration in the on-going development of live description services.

Apple’s lead in incorporating accessibility features in mainstream devices was appreciated by participants, evident in a strong preference for iOS systems in Australia. However, iOS affordability was a problem, particularly in Malaysia and so app development for both Apple and Android systems is needed.

### Strengths and limitations

The two country comparison provided an alternative means of triangulation to the two-phase evaluation framework proposed by Boland et al. (2014), where Phase 1 involves usability experts and Phase 2 involves end-user consultations. The Malaysian and Australian cohorts were small, unequal in size, and their heterogeneous make-up intentionally reflected the diverse nature, circumstances, and ages of O&M clientele. The online survey format enabled wider recruitment, but less control over who might respond, so the Australian cohort is loosely defined and possibly includes Australians living or travelling overseas. This eclectic approach to data collection captured a wide range of technologies used in O&M pre-COVID-19 and some cultural comparisons; it identified salient national differences in O&M practice, and priorities for any travel technologies used with low vision or blindness. However, results are suggestive rather than conclusive about the two cohorts. There were gaps in the Malaysian dataset as the survey questions were expanded for Australian administration; we did not reach saturation (Charmaz, 2014) with either cohort and this small, diverse dataset limited our statistical comparisons.

The 20%–22% of people with no light perception in both cohorts was double the 10% estimated in Australian O&M caseloads (Ah Tong et al., 2015) so despite small cohorts, the preferences of blind travellers were well represented in the study. However, recruitment strategies were biased towards people who already use O&M technologies, and little is yet known about reluctant O&M technology users in either country. Our parallel study investigating tech-use with O&M professionals confirmed that professionals have much to learn from the expertise of O&M clients evident in this study.

## Conclusion

This is the first study investigating O&M clients’ technology use in Australia and Malaysia. It shows that before COVID-19, participants in both countries were keenly interested in O&M technologies, and had already shifted away from using specialist travel aids to support their mobility. The unembellished primary mobility aids – human guide, long cane, and guide dog – continue to work well, and smartphone apps seemed able to provide the additional real-time information that O&M clients need to travel solo more confidently. The on-going value of real-time human assistance was evident in reports of helpful direct encounters during travel, and the uptake of live description services.

However, there are still significant barriers to access. In Malaysia, environmental streamlining, affordable long canes, and comprehensive training for O&M specialists are priorities to support independent travel for people with low vision or blindness. Australian pathways are more fluid, with information technologies now becoming integrated into urban design, but internet connection can be a problem.

O&M clients in both Australia and Malaysia valued affordable, synchronised, mainstream technologies with accurate, reliable data, and detailed pedestrian mapping as necessary foundations for other O&M technology developments. O&M technologies need to give prompt, reliable information to support travel in unfamiliar or less populated places.

This study, with its commitment to co-design, honours the call of disability advocates: nothing about us without us. It provides a user-centred evidence-base for development of environmental, mainstream, and specialised travel technologies by individual and industry innovators, university students, and design teams. It can also inform decisions about policy, research, development, commercialisation, and allocation of technologies and training programmes by national and local governments, urban designers, funding bodies, and O&M service providers.

## Supplemental Material

sj-pdf-1-jvi-10.1177_02646196211019070 – Supplemental material for Self-reported use of technology by orientation and mobility clients in Australia and Malaysia before the COVID-19 pandemic Click here for additional data file. Supplemental material, sj-pdf-1-jvi-10.1177_02646196211019070 for Self-reported use of technology by orientation and mobility clients in Australia and Malaysia before the COVID-19 pandemic by Lil Deverell, Jahar Bhowmik, Abdullah Al Mahmud, Bee Theng Lau, Fakir M Amirul Islam, Suku Sukunesan, Chris McCarthy and Denny Meyer in The British Journal of Visual Impairment

sj-pdf-2-jvi-10.1177_02646196211019070 – Supplemental material for Self-reported use of technology by orientation and mobility clients in Australia and Malaysia before the COVID-19 pandemic Click here for additional data file. Supplemental material, sj-pdf-2-jvi-10.1177_02646196211019070 for Self-reported use of technology by orientation and mobility clients in Australia and Malaysia before the COVID-19 pandemic by Lil Deverell, Jahar Bhowmik, Abdullah Al Mahmud, Bee Theng Lau, Fakir M Amirul Islam, Suku Sukunesan, Chris McCarthy and Denny Meyer in The British Journal of Visual Impairment
