# Supplementary material for: Self-reported use of technology by orientation and mobility clients in Australia and Malaysia before the COVID-19 pandemic
Source: Br J Vis Impair. 2023 Jan;41(1):33–48. doi: 10.1177/02646196211019070 (PMC8185563; doi:10.1177/02646196211019070)
Supplement: sj-pdf-1-jvi-10.1177_02646196211019070 – Supplemental material for Self-reported use of technology by orientation and mobility clients in Australia and Malaysia before the COVID-19 pandemic [file sj-pdf-1-jvi-10.1177_02646196211019070.pdf]

Supplement: Malaysian and Australian Technology Surveys for O&M Clients

**Technology survey for O&M clients**<br>Date: _________________<br>Venue: ______________________________	**Kajian teknologi untuk pelanggan O & M**<br>Tarikh: _________________<br>Tempat: _______________________________
Q1. Gender<br>○ Male<br>◉ Female	Q1. Jantina<br>○ Lelaki<br>○ Perempuan
Q2. Age<br>○ Less than 10<br>○ 10-19<br>○ 20-29<br>○ 30-39<br>○ 40-49<br>○ 50-59<br>○ 60-69<br>○ 70 +	Q2. Umur<br>○ < 10<br>○ 10-19<br>○ 20-29<br>○ 30-39<br>○ 40-49<br>○ 50-59<br>○ 60-69<br>○ 70 +
Q3. How would you describe your sight?<br>○ No light perception<br>○ Low vision and legally blind (eligible for pension)<br>○ Low vision, but not eligible for pension<br>○ Full vision	Q3. Bagaimana anda menggambarkan pandangan anda?<br>○ Tiada persepsi ringan<br>○ Visi rendah dan buta secara sah (layak untuk pencen)<br>○ Penglihatan yang rendah, tetapi tidak layak untuk pencen<br>○ Visi penuh
Q4. How do you travel beyond home?<br>☐ Long cane<br>☐ Dog guide<br>☐ Sighted guide<br>☐ Taxi/Uber<br>☐ Public transport<br>☐ Private car<br>☐ Wheelchair<br>☐ Scooter<br>☐ Bicycle<br>☐ Other. Please explain:	Q4. Bagaimanakah perjalanan anda ke luar rumah?<br>○ Rotan panjang<br>○ Panduan anjing<br>○ Panduan yang dipertimbangkan<br>○ Teksi / Uber<br>○ Pengangkutan awam<br>○ Kereta persendirian<br>○ Kerusi roda<br>○ Skuter<br>○ Basikal<br>○ Lain-lain. Sila jelaskan:

Q5. Do you use any of the following devices? (can choose more than one)<br><br>☐ Mobile phone/s. Make and model:<br>☐ Tablet. Make and model:<br>☐ Portable braille notetaker. Make and model:<br>☐ Laptop computer. Make and model:<br>☐ Desktop computer. Make and model:<br>☐ Standalone GPS. Make and model:<br>☐ Standalone OCR (optical character recognition) device. Make and model:<br>☐ CCTV (closed circuit television). Make and model:<br>☐ Personal activity monitor (e.g, Fitbit, SmartWatch). Make and model:<br>☐ Handheld sonar (e.g., Miniguide). Make and model:<br>☐ Sonar built into another device (e.g., Ultracane). Make and model:<br>☐ Barcode Reader. Make and model:<br>☐ Other. Please describe:	Q5. Adakah anda menggunakan mana-mana peranti berikut? (boleh memilih lebih daripada satu)<br><br>○ Telefon bimbit. Buat dan model:<br>○ Tablet. Buat dan model:<br>○ Pembuat braille mudah alih. Buat dan model:<br>○ Komputer riba. Buat dan model:<br>○ Komputer meja. Buat dan model:<br>○ GPS berdiri sendiri. Buat dan model:<br>○ Standalone OCR (pengiktirafan aksara optik). Buat dan model:<br>○ CCTV (televisyen litar tertutup). Buat dan model:<br>○ Memantau aktiviti peribadi (contohnya, Fitbit, SmartWatch). Buat dan model:<br>○ Sonar pegangan tangan (cth., Miniguide). Buat dan model:<br>○ Sonar dibina ke peranti lain (cth., Ultracane). Buat dan model:<br>○ Pembaca kod bar. Buat dan model:<br>○ Lain-lain. Sila nyatakan:
Q6. What formats do you use to support your travel? (can choose more than one)<br><br>☐ Print on paper<br>☐ Screen magnifier (zoom)<br>☐ Screen reader (voice-over)<br>☐ Voice recorder<br>☐ Braille<br>☐ I plan and/or travel with someone else<br>☐ Other. Please explain	Q6. Format apa yang anda gunakan untuk menyokong perjalanan anda? (boleh memilih lebih daripada satu)<br><br>○ Cetak pada kertas<br>○ Pembesar skrin (zum)<br>○ Pembaca skrin (suara)<br>○ Rakaman suara<br>○ Braille<br>○ Saya merancang dan / atau bepergian dengan orang lain<br>○ Lain-lain. Sila jelaskan
Q7. Which apps do you use to plan or carry out travel?<br>☐ Please list:<br>☐ I don’t use apps.	Q7. Aplikasi mana yang anda gunakan untuk merancang atau menjalankan perjalanan?<br><br>○ Sila nyatakan:<br>○ Saya tidak menggunakan aplikasi.

Q8. What features do you particularly like in the apps you use?<br>☐ Please explain.<br>☐ I don't use apps.	Q8. Ciri-ciri apa yang anda suka terutamanya dalam aplikasi yang anda gunakan?<br>○ Sila jelaskan.<br>○ Saya tidak menggunakan aplikasi.
Q9. Do you have access to technology training for travel purposes?<br>☐ Yes. Please describe:<br>☐ I would like more training. Please describe:<br>☐ No	Q9. Adakah anda mempunyai akses kepada latihan teknologi untuk tujuan perjalanan?<br>○ Ya. Sila nyatakan:<br>○ Saya ingin lebih banyak latihan. Sila nyatakan:<br>○ Tidak
Q10. Do you have ideas about how technology could be developed or enhanced to support your travel?<br>○ Yes. Please explain:<br>○ No.	Q10. Adakah anda mempunyai idea tentang bagaimana teknologi dapat dibangunkan atau dipertingkatkan untuk menyokong perjalanan anda?<br>○ Ya. Sila jelaskan:<br>○ Tidak.
Q11. Are you happy to be contacted by a Swinburne researcher to discuss your technology ideas?<br>☐ Your name:<br>☐ Best phone number:<br>☐ Best email address:	Q11. Adakah anda gembira dihubungi oleh penyelidik Swinburne untuk membincangkan idea teknologi anda?<br>○ Nama awak:<br>○ Nombor telefon terbaik:<br>○ Alamat e-mel terbaik:
Q12. How did you respond to this survey?<br>☐ Independently<br>☐ With some assistance	Q12. Bagaimana anda bertindak balas terhadap tinjauan ini?<br>○ Bebas<br>○ Dengan bantuan

Technology survey for O&M clients (Australia)

Q1 **Technology for O&M**

Thank you for taking part in this survey. It will take about ten minutes to complete - there are 19 questions.

We are interested in how people with low vision or blindness use technology to support your orientation and mobility (O&M), both in your planning and while you travel. We are also interested in your ideas about new or modified technology that could improve your O&M experience whether you're out alone, travelling with others, or working with an O&M specialist.

The survey results will be shared with technology developers, providers and trainers, as well as through O&M service providers to clients, O&M specialists and dog guide mobility instructors.

xxx
Researching Orientation and Mobility

Q2 What is your gender

- ◯ Male
- ◯ Female
- ◯ Other

Q3 What is your age?

- ◯ Less than 10
- ◯ 10-19
- ◯ 20-29
- ◯ 30-39
- ◯ 40-49
- ◯ 50-59
- ◯ 60-69
- ◯ 70 +

Q4 Where do you live?

▼ Australia ... Other

Q5 Do you use technology in a language other than English?

- ◯ No
- ◯ Yes - Please list language/s ____

Q6 How much sight do you have?

- ○ No light perception - totally blind
- ○ Low vision, eligible for a pension or allowance (legally blind)
- ○ Low vision, but not eligible for a pension
- ○ Full vision

Q7 Have you used any physical mobility aids in the past year? You can choose more than one.

- ☐ No aids
- ☐ No aids, but I need to go with a companion sometimes
- ☐ Human guide (holding on)
- ☐ Dog guide / guide dog
- ☐ Long cane
- ☐ Support cane / walking stick
- ☐ Identification cane / symbol cane
- ☐ Walking frame
- ☐ Manual wheelchair
- ☐ Powerchair
- ☐ Motorised mobility scooter
- ☐ Other: ____

Q8 What kinds of transport have you used in the past year? You can choose more than one.

- ☐ Private car
- ☐ Motor bike
- ☐ Taxi
- ☐ Uber
- ☐ Bus
- ☐ Train
- ☐ Tram
- ☐ Ferry / water taxi

- ☐ Bicycle / tricycle / tandem
- ☐ Foot scooter / skateboard / skates
- ☐ Aeroplane
- ☐ Cruise ship / boat
- ☐ Other: ____

Q9 What reading/writing formats do you use to access and manage travel information (e.g., timetables, addresses, shopping lists, maps or directions?) You can choose more than one.

- ☐ Regular print on paper
- ☐ Large print on paper
- ☐ Hand-held low vision aids (e.g., magnifier, monocular)
- ☐ Text on screen
- ☐ Screen magnifier / zoom technology
- ☐ Screen reader / voice-over technology
- ☐ Voice recorder - device or app
- ☐ Audio / radio
- ☐ Braille
- ☐ Someone else manages my travel information
- ☐ Other: ____

Q10 Do you use any of the following <u>devices</u>? You can choose more than one, and we'll ask about apps next.

- ☐ Telephone landline - list any modifications ________________________________
- ☐ Mobile phone/s - make and model: ____
- ☐ Tablet - make and model: ____
- ☐ Laptop or desktop computer - make and model: _____________________________
- ☐ Portable braille note-taker - make and model: ______________________________
- ☐ Standalone OCR (optical character recognition) - make and model: ____________
- ☐ CCTV (closed-circuit television) - make and model: _________________________

- ☐ Standalone GPS (e.g. Trekker) - make and model: ___________________________
- ☐ Personal activity monitor (e.g. Fitbit, SmartWatch) - make and model: ____________
- ☐ Handheld sonar (e.g., Miniguide) - make and model: __________________________
- ☐ Sonar built into another device (e.g., Ultracane) - make and model: ______________
- ☐ Barcode reader - make and model: ____
- ☐ Other: ____

Q11 Which <u>apps or websites</u> do you use to support your travel? Consider journey planning and directions, making bookings, recording lists or details, getting help if lost, and entertainment on the way.

- ○ Please list: ____
- ○ Not applicable

Q12 What are your priorities when choosing new technologies to support your travel?

- ○ Please explain: ____
- ○ Not applicable

Q13 Are there devices or technologies for travel that you have, but don't use?

- ○ No
- ○ Yes - Please explain ____

Q14 How have you learned about technology? You can choose more than one.

- ☐ Trial and error
- ☐ Friends / family
- ☐ Colleagues, as needed
- ☐ During formal training for a qualification (e.g., certificate, diploma, degree)
- ☐ Short courses, face-to-face
- ☐ Short courses, online
- ☐ Online Help (e.g., websites, manuals, chatrooms)
- ☐ A paid expert / consultant
- ☐ Other ____

Q15 How would you rate your technology skills? You can choose more than one.

- ☐ I help to train others to use technology.
- ☐ I'm good at technology. I work it out fairly quickly.
- ☐ I have enough technology skills to keep me going.
- ☐ I have some technology skills, but I need more.
- ☐ I use technology, but I don't much enjoy it.
- ☐ I avoid using technology wherever possible.
- ☐ I can access technology training or help when I need it. Please list training providers: ____
- ☐ I have limited access to training or help with technology.
- ☐ I would like more technology training. Please describe: ______________________
- ☐ Not applicable

Q16 Do you have ideas about how technology could be developed or enhanced to support your travel?

- ○ No
- ○ Yes. Please explain: ____

Q17 If you are happy to discuss your technology ideas with a Swinburne researcher, please give your contact details:

- ☐ Name: ____
- ☐ Email: ____
- ☐ Phone: ____

Q18 Is there anything else you want to tell us about developing good technologies for travel?

- ○ No
- ○ Yes - Please explain ____

Q19 How did you respond to this survey?

- ○ Independently
- ○ With some assistance
