# Supplementary material for: Self-reported use of technology by orientation and mobility clients in Australia and Malaysia before the COVID-19 pandemic
Source: Br J Vis Impair. 2023 Jan;41(1):33–48. doi: 10.1177/02646196211019070 (PMC8185563; doi:10.1177/02646196211019070)

Supplement 2: Apps and websites used by O&M Clients in Australia and Malaysia to support travel, before COVID-19

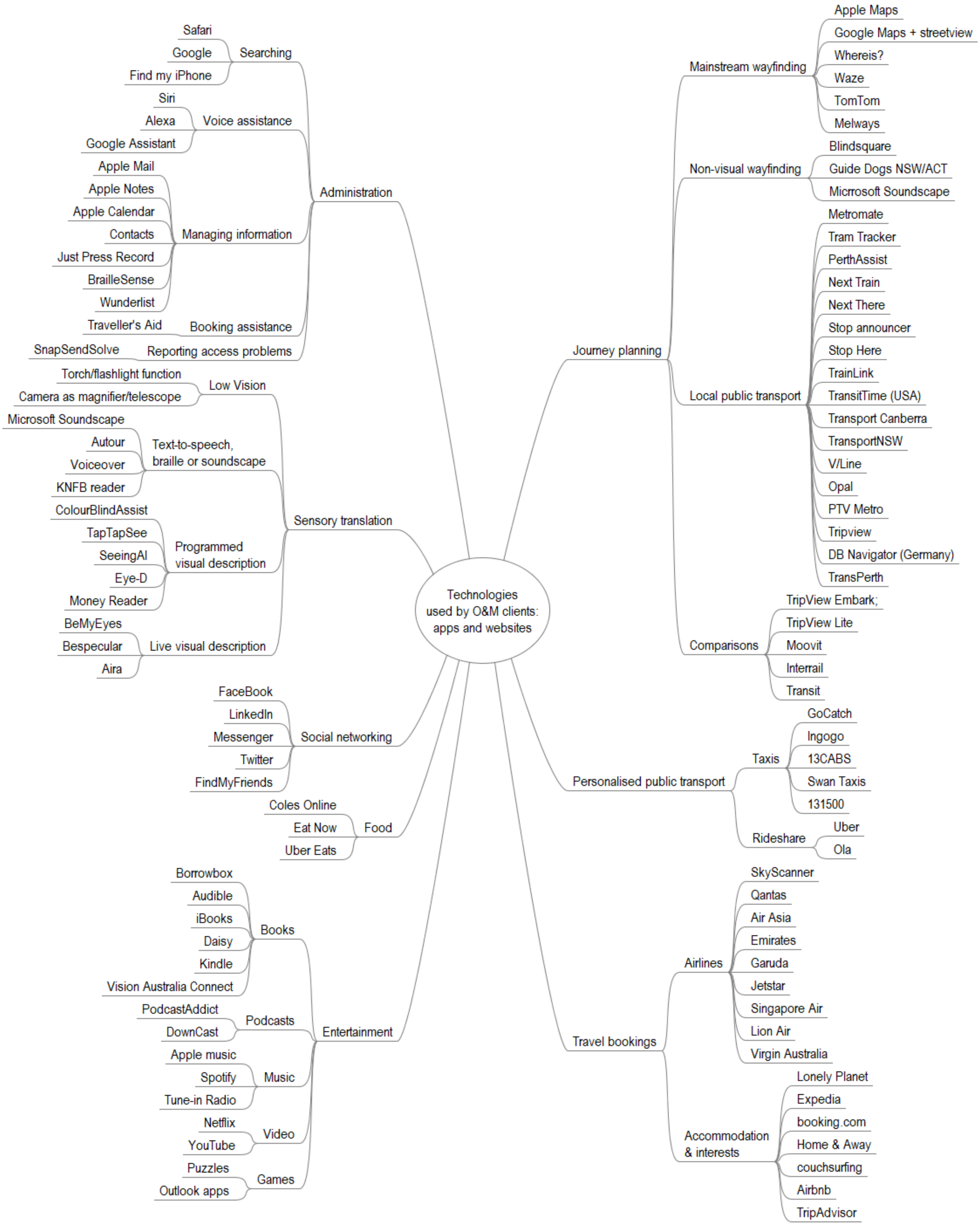

Supplement: sj-pdf-2-jvi-10.1177_02646196211019070 – Supplemental material for Self-reported use of technology by orientation and mobility clients in Australia and Malaysia before the COVID-19 pandemic [file sj-pdf-2-jvi-10.1177_02646196211019070.pdf]
